# Does the correlation between informant ratings of function and cognitive assessment scores differ based on informant gender?

**DOI:** 10.1002/alz.091544

**Published:** 2025-01-03

**Authors:** Megan Stradtman, Alyssa N. De Vito, Ashley N. Price, Jennifer Strenger, Louisa I. Thompson, Stuart Sinoff, Peter J. Snyder, Jessica Alber

**Affiliations:** ^1^ Butler Hospital Memory and Aging Program, Providence, RI USA; ^2^ Alpert Medical School of Brown University, Providence, RI USA; ^3^ University of Rhode Island, Kingston, RI USA; ^4^ Bryn Mawr College, Bryn Mawr, PA USA; ^5^ BayCare Health, Clearwater, FL USA; ^6^ George & Anne Ryan Institute for Neuroscience, Kingston, RI USA

## Abstract

**Background:**

Informant reports can complement standardized cognitive assessment and improve accuracy of dementia diagnosis. Although informant reports correlate moderately with objective measures of decline, the influence of informant factors, such as gender, on these relationships is unclear. This study assessed the hypothesis that informant gender would emerge as an independent predictor in the relationship between informant ratings of cognitive function (i.e., Clinical Dementia Rating Scale) and cognitive assessment scores.

**Method:**

204 adults aged 55‐80 (124 F, 80 M) completed the CDR with study partners (106 F, 95 M). Participants completed the Repeatable Battery for the Assessment of Neuropsychological Status (RBANS) and CDR at screening, and the Free and Cued Selective Reminding Test (FCSRT) within 12 weeks of screening. RBANS delayed memory index scores were available for 203 participants, and the RBANS total scores for 200 participants. 148 participants had FCSRT immediate free recall and delayed free recall scores available. Linear regression analyses were used to evaluate whether informant gender significantly predicted the relationship between the CDR sum of boxes (SOB) and: RBANS total score, RBANS delayed memory index score, FCRST immediate recall score, and FCRST delayed recall score, while controlling for age and education.

**Result:**

The CDR SOB was negatively associated with RBANS total score (R^2^ = .547, β = ‐.511, *p*< .001), RBANS delayed memory index score (R^2^ = .640, β = ‐.643, *p*< .001), FCRST immediate free recall (R^2^ = .220, β = ‐.162, *p* = .025), and FCRST delayed free recall (R^2^ = .667, β = ‐.646, *p*<; .001). Informant gender was not associated with any cognitive outcome (*p*’s> .05).

**Conclusion:**

These results did not support the hypothesis that participants with female study partners would show a stronger correlation between informant ratings of cognitive function and cognitive assessment scores. Future work will: 1) use qualitative analysis to investigate potential gender differences in informant reporting and 2) examine additional informant‐related factors that may impact the relationship between informant ratings and neuropsychological testing such as informant level of education, culture, and family history and/or experience with dementia.

